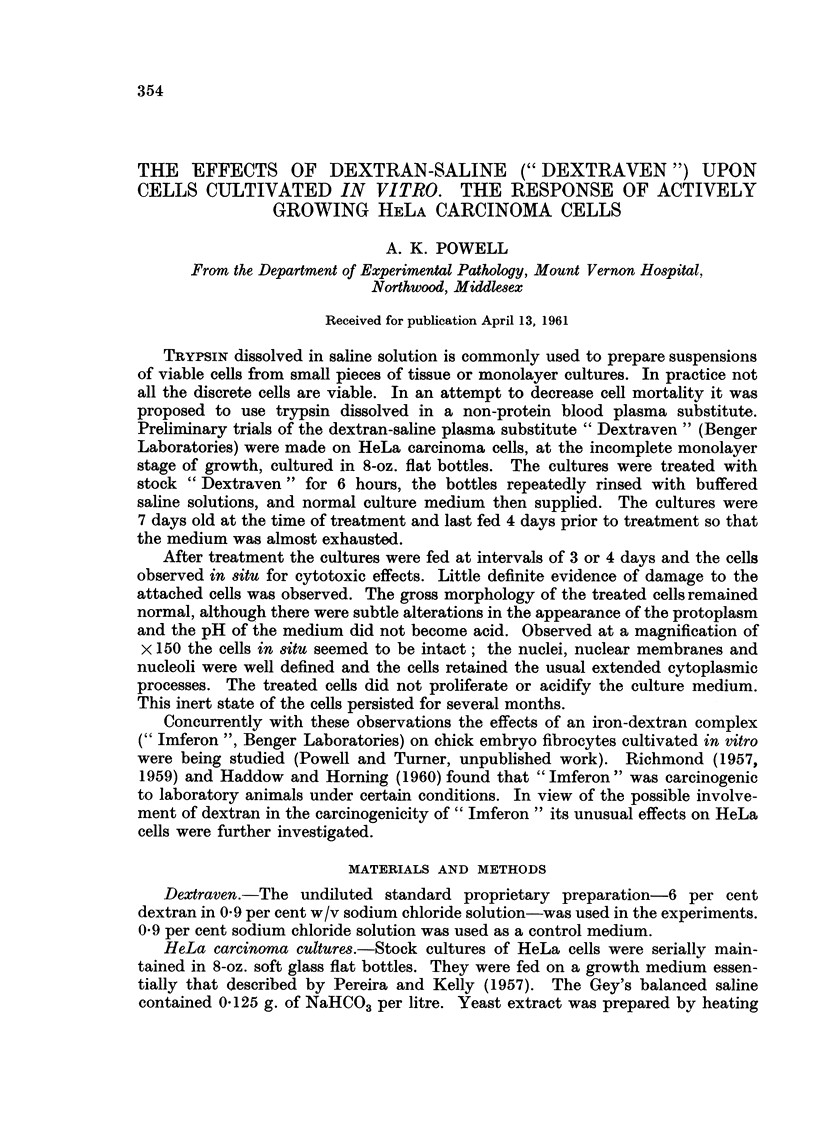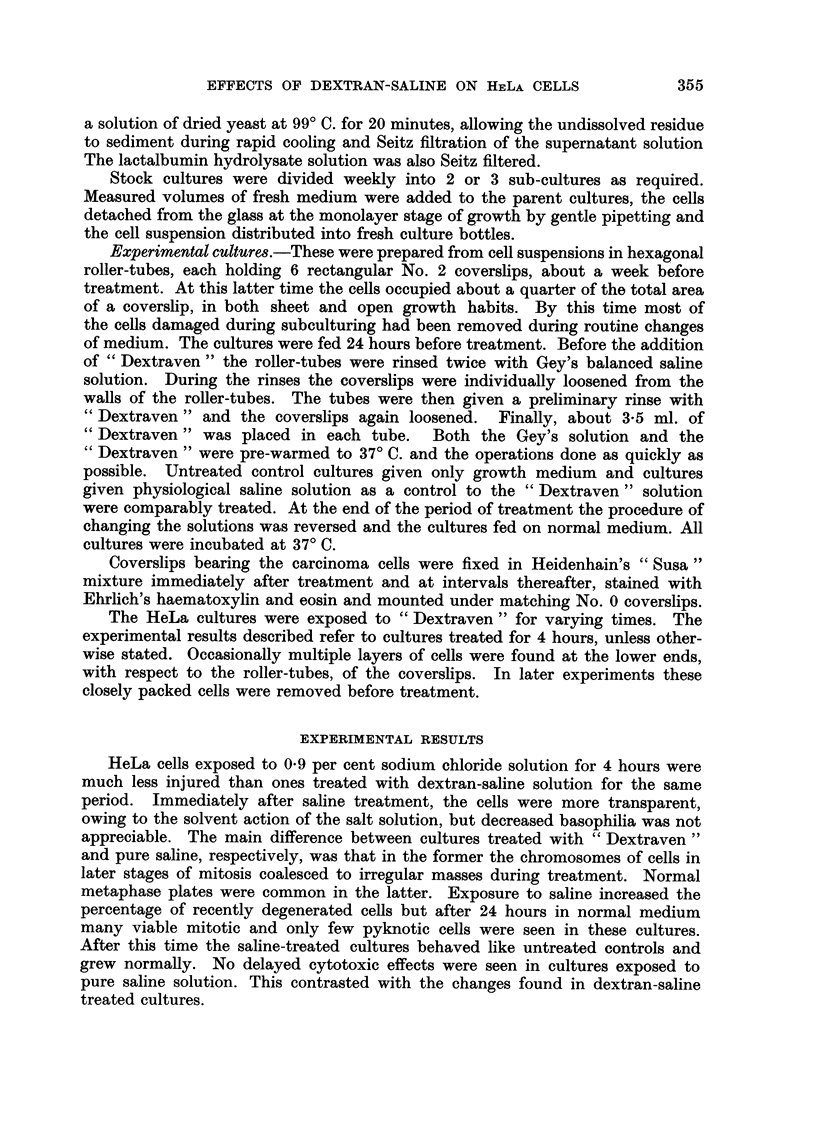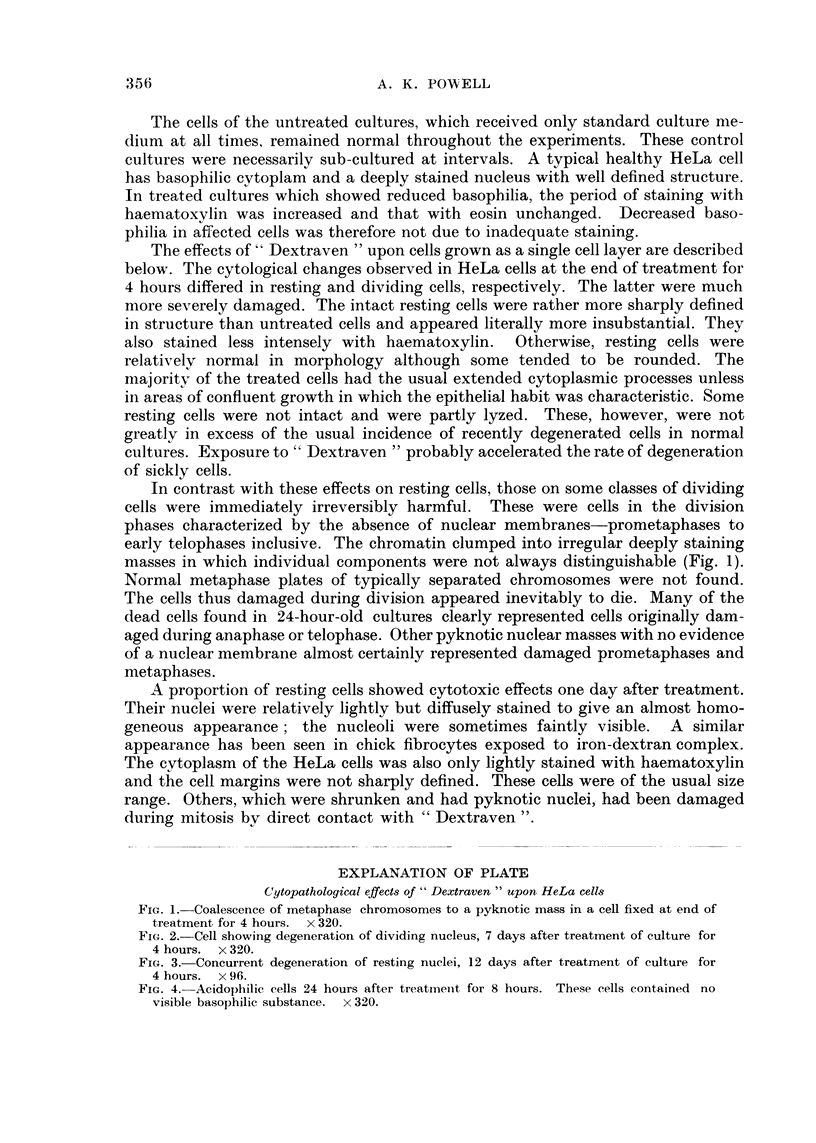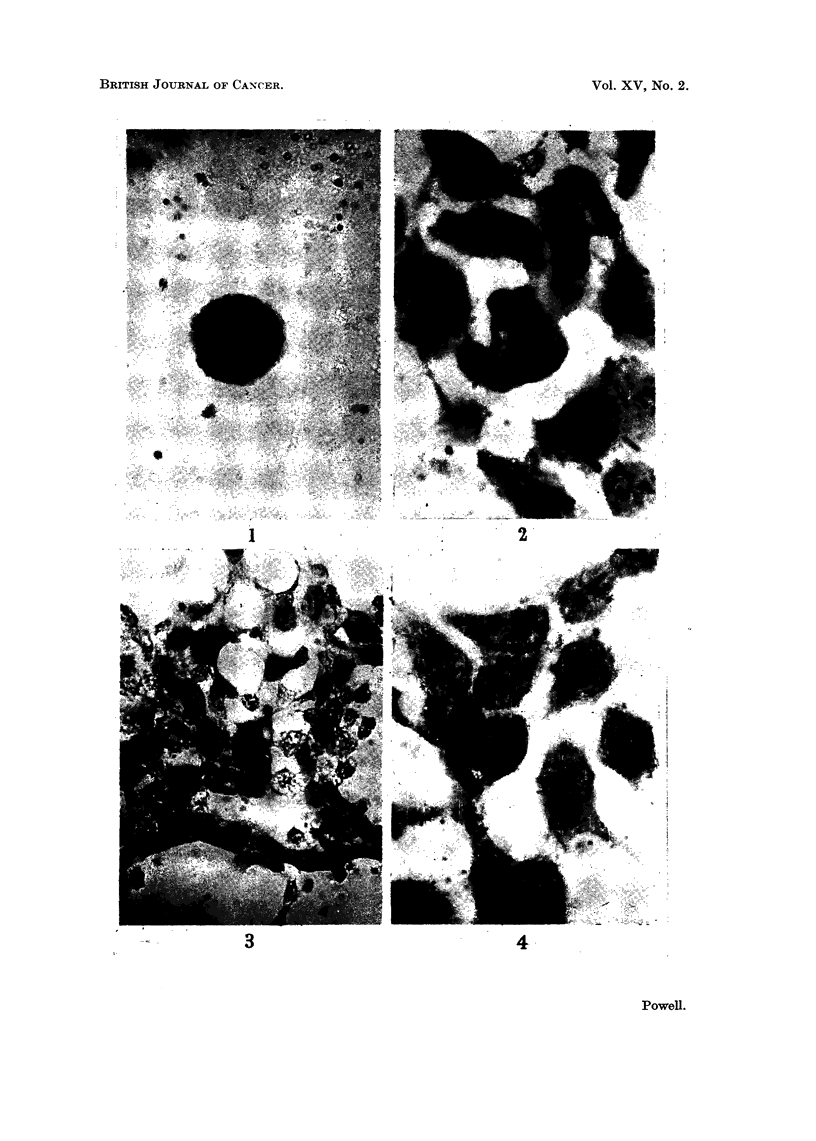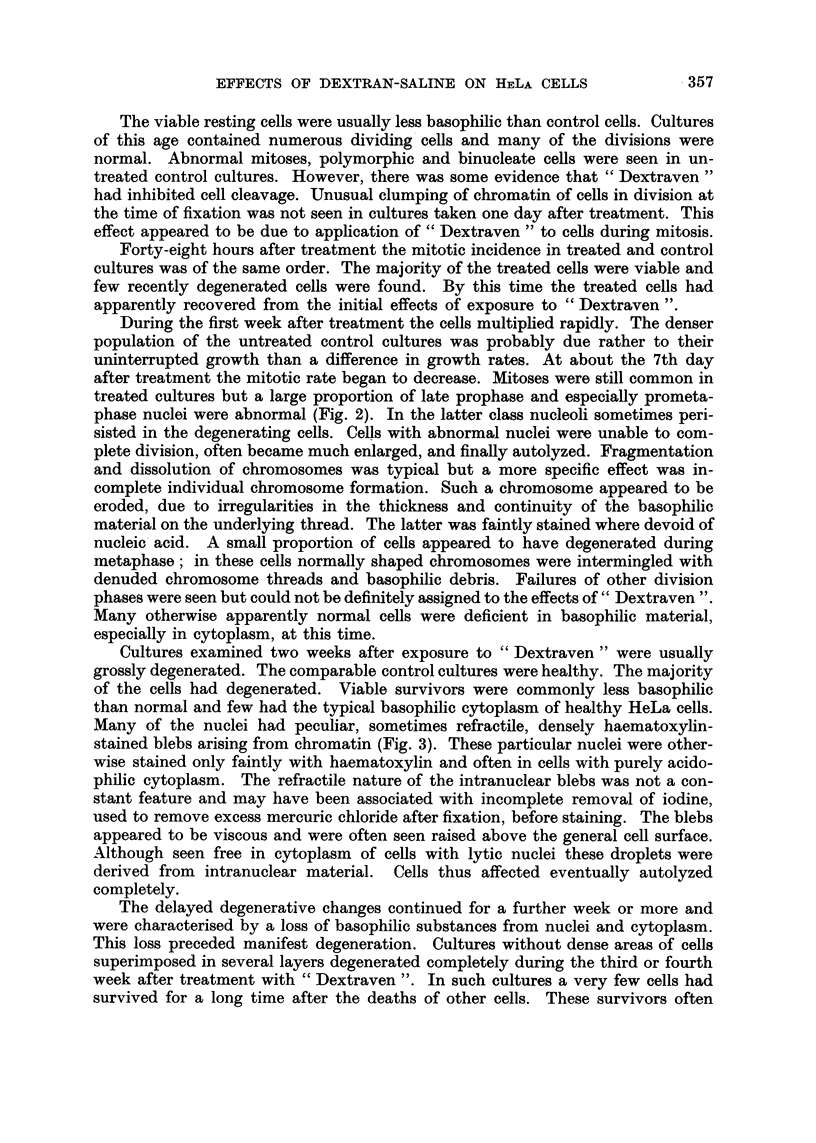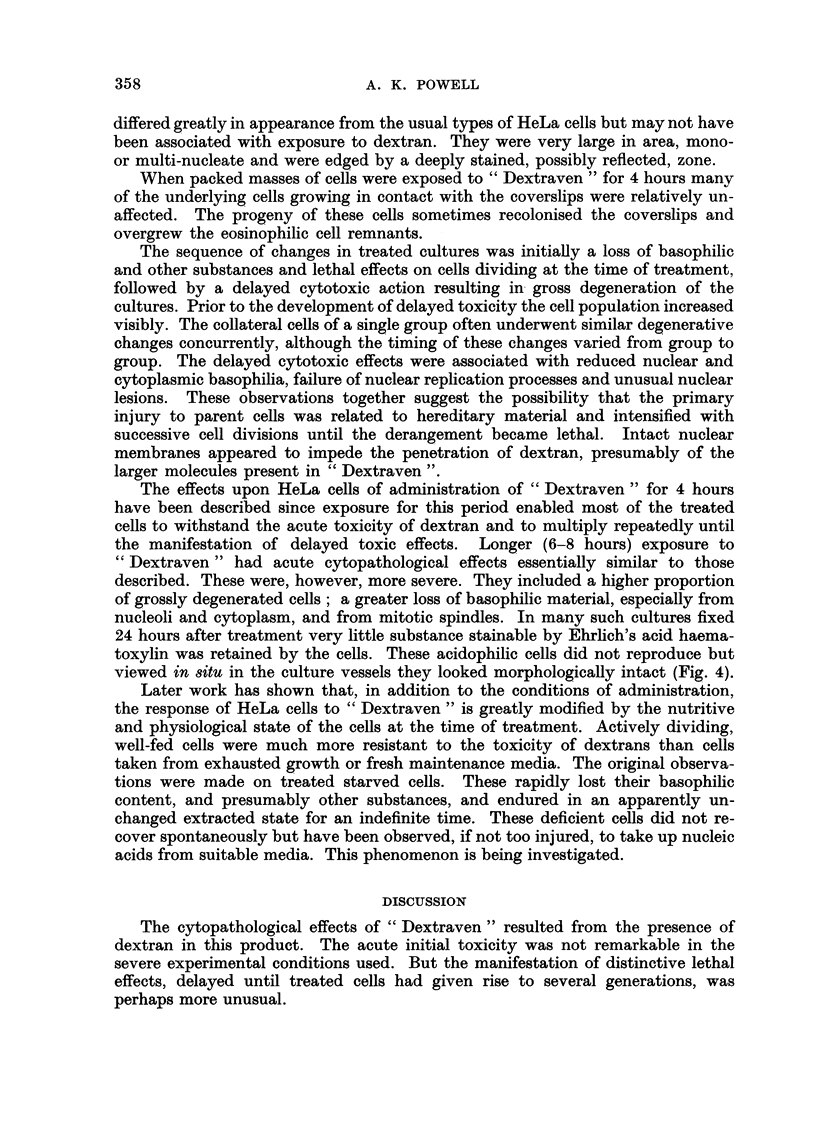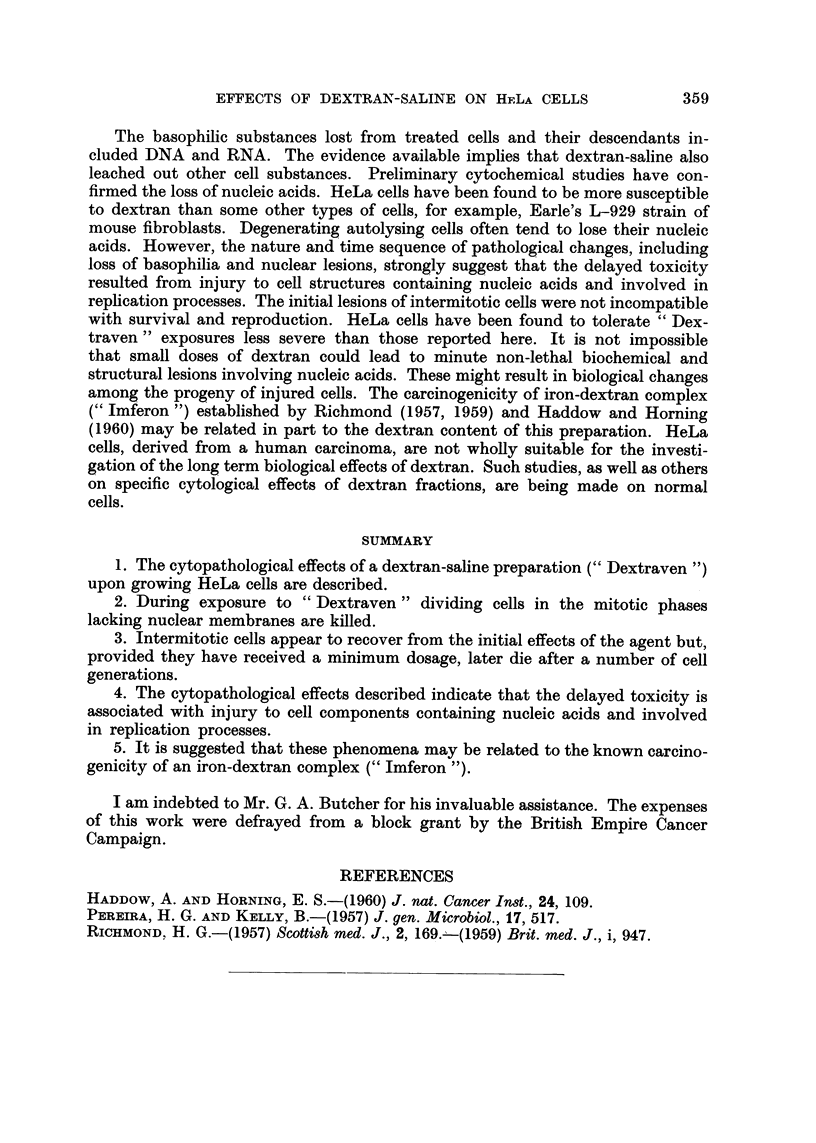# The Effects of Dextran-saline (“ Dextraven ”) upon Cells Cultivated in vitro. The Response of Actively Growing HeLa Carcinoma Cells

**DOI:** 10.1038/bjc.1961.45

**Published:** 1961-06

**Authors:** A. K. Powell

## Abstract

**Images:**


### THE EFFECTS OF DEXTRAN-SALINE (" DEXTRAVEN ") UPON CELLS CULTIVATED *IN VITRO*. THE RESPONSE OF ACTIVELY GROWING HeLa CARCINOMA CELLS

A. K. POWELL

*From the Department of Experimental Pathology, Mount Vernon Hospital, Northwood, Middlesex*

Received for publication April 13, 1961

TRYPSIN dissolved in saline solution is commonly used to prepare suspensions of viable cells from small pieces of tissue or monolayer cultures. In practice not all the discrete cells are viable. In an attempt to decrease cell mortality it was proposed to use trypsin dissolved in a non-protein blood plasma substitute. Preliminary trials of the dextran-saline plasma substitute " Dextraven " (Benger Laboratories) were made on HeLa carcinoma cells, at the incomplete monolayer stage of growth, cultured in 8-oz. flat bottles. The cultures were treated with stock " Dextraven " for 6 hours, the bottles repeatedly rinsed with buffered saline solutions, and normal culture medium then supplied. The cultures were 7 days old at the time of treatment and last fed 4 days prior to treatment so that the medium was almost exhausted.

After treatment the cultures were fed at intervals of 3 or 4 days and the cells observed *in situ* for cytotoxic effects. Little definite evidence of damage to the attached cells was observed. The gross morphology of the treated cells remained normal, although there were subtle alterations in the appearance of the protoplasm and the pH of the medium did not become acid. Observed at a magnification of $\times 150$ the cells *in situ* seemed to be intact ; the nuclei, nuclear membranes and nucleoli were well defined and the cells retained the usual extended cytoplasmic processes. The treated cells did not proliferate or acidify the culture medium. This inert state of the cells persisted for several months.

Concurrently with these observations the effects of an iron-dextran complex (" Imferon ", Benger Laboratories) on chick embryo fibrocytes cultivated *in vitro* were being studied (Powell and Turner, unpublished work). Richmond (1957, 1959) and Haddow and Horning (1960) found that " Imferon " was carcinogenic to laboratory animals under certain conditions. In view of the possible involvement of dextran in the carcinogenicity of " Imferon " its unusual effects on HeLa cells were further investigated.

### MATERIALS AND METHODS

*Dextraven.*—The undiluted standard proprietary preparation—6 per cent dextran in 0·9 per cent w/v sodium chloride solution—was used in the experiments. 0·9 per cent sodium chloride solution was used as a control medium.

*HeLa carcinoma cultures.*—Stock cultures of HeLa cells were serially maintained in 8-oz. soft glass flat bottles. They were fed on a growth medium essentially that described by Pereira and Kelly (1957). The Gey's balanced saline contained 0·125 g. of $NaHCO_3$ per litre. Yeast extract was prepared by heating a solution of dried yeast at 99° C. for 20 minutes, allowing the undissolved residue to sediment during rapid cooling and Seitz filtration of the supernatant solution The lactalbumin hydrolysate solution was also Seitz filtered.

Stock cultures were divided weekly into 2 or 3 sub-cultures as required. Measured volumes of fresh medium were added to the parent cultures, the cells detached from the glass at the monolayer stage of growth by gentle pipetting and the cell suspension distributed into fresh culture bottles.

*Experimental cultures.*—These were prepared from cell suspensions in hexagonal roller-tubes, each holding 6 rectangular No. 2 coverslips, about a week before treatment. At this latter time the cells occupied about a quarter of the total area of a coverslip, in both sheet and open growth habits. By this time most of the cells damaged during subculturing had been removed during routine changes of medium. The cultures were fed 24 hours before treatment. Before the addition of " Dextraven " the roller-tubes were rinsed twice with Gey's balanced saline solution. During the rinses the coverslips were individually loosened from the walls of the roller-tubes. The tubes were then given a preliminary rinse with " Dextraven " and the coverslips again loosened. Finally, about 3·5 ml. of " Dextraven " was placed in each tube. Both the Gey's solution and the " Dextraven " were pre-warmed to 37° C. and the operations done as quickly as possible. Untreated control cultures given only growth medium and cultures given physiological saline solution as a control to the " Dextraven " solution were comparably treated. At the end of the period of treatment the procedure of changing the solutions was reversed and the cultures fed on normal medium. All cultures were incubated at 37° C.

Coverslips bearing the carcinoma cells were fixed in Heidenhain's " Susa " mixture immediately after treatment and at intervals thereafter, stained with Ehrlich's haematoxylin and eosin and mounted under matching No. 0 coverslips.

The HeLa cultures were exposed to " Dextraven " for varying times. The experimental results described refer to cultures treated for 4 hours, unless otherwise stated. Occasionally multiple layers of cells were found at the lower ends, with respect to the roller-tubes, of the coverslips. In later experiments these closely packed cells were removed before treatment.

### EXPERIMENTAL RESULTS

HeLa cells exposed to 0·9 per cent sodium chloride solution for 4 hours were much less injured than ones treated with dextran-saline solution for the same period. Immediately after saline treatment, the cells were more transparent, owing to the solvent action of the salt solution, but decreased basophilia was not appreciable. The main difference between cultures treated with " Dextraven " and pure saline, respectively, was that in the former the chromosomes of cells in later stages of mitosis coalesced to irregular masses during treatment. Normal metaphase plates were common in the latter. Exposure to saline increased the percentage of recently degenerated cells but after 24 hours in normal medium many viable mitotic and only few pyknotic cells were seen in these cultures. After this time the saline-treated cultures behaved like untreated controls and grew normally. No delayed cytotoxic effects were seen in cultures exposed to pure saline solution. This contrasted with the changes found in dextran-saline treated cultures.

The cells of the untreated cultures, which received only standard culture medium at all times, remained normal throughout the experiments. These control cultures were necessarily sub-cultured at intervals. A typical healthy HeLa cell has basophilic cytoplam and a deeply stained nucleus with well defined structure. In treated cultures which showed reduced basophilia, the period of staining with haematoxylin was increased and that with eosin unchanged. Decreased basophilia in affected cells was therefore not due to inadequate staining.

The effects of " Dextraven " upon cells grown as a single cell layer are described below. The cytological changes observed in HeLa cells at the end of treatment for 4 hours differed in resting and dividing cells, respectively. The latter were much more severely damaged. The intact resting cells were rather more sharply defined in structure than untreated cells and appeared literally more insubstantial. They also stained less intensely with haematoxylin. Otherwise, resting cells were relatively normal in morphology although some tended to be rounded. The majority of the treated cells had the usual extended cytoplasmic processes unless in areas of confluent growth in which the epithelial habit was characteristic. Some resting cells were not intact and were partly lyzed. These, however, were not greatly in excess of the usual incidence of recently degenerated cells in normal cultures. Exposure to " Dextraven " probably accelerated the rate of degeneration of sickly cells.

In contrast with these effects on resting cells, those on some classes of dividing cells were immediately irreversibly harmful. These were cells in the division phases characterized by the absence of nuclear membranes—prometaphases to early telophases inclusive. The chromatin clumped into irregular deeply staining masses in which individual components were not always distinguishable (Fig. 1). Normal metaphase plates of typically separated chromosomes were not found. The cells thus damaged during division appeared inevitably to die. Many of the dead cells found in 24-hour-old cultures clearly represented cells originally damaged during anaphase or telophase. Other pyknotic nuclear masses with no evidence of a nuclear membrane almost certainly represented damaged prometaphases and metaphases.

A proportion of resting cells showed cytotoxic effects one day after treatment. Their nuclei were relatively lightly but diffusely stained to give an almost homogeneous appearance ; the nucleoli were sometimes faintly visible. A similar appearance has been seen in chick fibrocytes exposed to iron-dextran complex. The cytoplasm of the HeLa cells was also only lightly stained with haematoxylin and the cell margins were not sharply defined. These cells were of the usual size range. Others, which were shrunken and had pyknotic nuclei, had been damaged during mitosis by direct contact with " Dextraven ".

### EXPLANATION OF PLATE

*Cytopathological effects of " Dextraven " upon HeLa cells*

FIG. 1.—Coalescence of metaphase chromosomes to a pyknotic mass in a cell fixed at end of treatment for 4 hours. ×320.

FIG. 2.—Cell showing degeneration of dividing nucleus, 7 days after treatment of culture for 4 hours. ×320.

FIG. 3.—Concurrent degeneration of resting nuclei, 12 days after treatment of culture for 4 hours. ×96.

FIG. 4.—Acidophilic cells 24 hours after treatment for 8 hours. These cells contained no visible basophilic substance. ×320.

1 2 3 4

Powell.

The viable resting cells were usually less basophilic than control cells. Cultures of this age contained numerous dividing cells and many of the divisions were normal. Abnormal mitoses, polymorphic and binucleate cells were seen in untreated control cultures. However, there was some evidence that " Dextraven " had inhibited cell cleavage. Unusual clumping of chromatin of cells in division at the time of fixation was not seen in cultures taken one day after treatment. This effect appeared to be due to application of " Dextraven " to cells during mitosis.

Forty-eight hours after treatment the mitotic incidence in treated and control cultures was of the same order. The majority of the treated cells were viable and few recently degenerated cells were found. By this time the treated cells had apparently recovered from the initial effects of exposure to " Dextraven ".

During the first week after treatment the cells multiplied rapidly. The denser population of the untreated control cultures was probably due rather to their uninterrupted growth than a difference in growth rates. At about the 7th day after treatment the mitotic rate began to decrease. Mitoses were still common in treated cultures but a large proportion of late prophase and especially prometaphase nuclei were abnormal (Fig. 2). In the latter class nucleoli sometimes perisisted in the degenerating cells. Cells with abnormal nuclei were unable to complete division, often became much enlarged, and finally autolyzed. Fragmentation and dissolution of chromosomes was typical but a more specific effect was incomplete individual chromosome formation. Such a chromosome appeared to be eroded, due to irregularities in the thickness and continuity of the basophilic material on the underlying thread. The latter was faintly stained where devoid of nucleic acid. A small proportion of cells appeared to have degenerated during metaphase ; in these cells normally shaped chromosomes were intermingled with denuded chromosome threads and basophilic debris. Failures of other division phases were seen but could not be definitely assigned to the effects of " Dextraven ". Many otherwise apparently normal cells were deficient in basophilic material, especially in cytoplasm, at this time.

Cultures examined two weeks after exposure to " Dextraven " were usually grossly degenerated. The comparable control cultures were healthy. The majority of the cells had degenerated. Viable survivors were commonly less basophilic than normal and few had the typical basophilic cytoplasm of healthy HeLa cells. Many of the nuclei had peculiar, sometimes refractile, densely haematoxylin-stained blebs arising from chromatin (Fig. 3). These particular nuclei were otherwise stained only faintly with haematoxylin and often in cells with purely acidophilic cytoplasm. The refractile nature of the intranuclear blebs was not a constant feature and may have been associated with incomplete removal of iodine, used to remove excess mercuric chloride after fixation, before staining. The blebs appeared to be viscous and were often seen raised above the general cell surface. Although seen free in cytoplasm of cells with lytic nuclei these droplets were derived from intranuclear material. Cells thus affected eventually autolyzed completely.

The delayed degenerative changes continued for a further week or more and were characterised by a loss of basophilic substances from nuclei and cytoplasm. This loss preceded manifest degeneration. Cultures without dense areas of cells superimposed in several layers degenerated completely during the third or fourth week after treatment with " Dextraven ". In such cultures a very few cells had survived for a long time after the deaths of other cells. These survivors often differed greatly in appearance from the usual types of HeLa cells but may not have been associated with exposure to dextran. They were very large in area, mono- or multi-nucleate and were edged by a deeply stained, possibly reflected, zone.

When packed masses of cells were exposed to " Dextraven " for 4 hours many of the underlying cells growing in contact with the coverslips were relatively unaffected. The progeny of these cells sometimes recolonised the coverslips and overgrew the eosinophilic cell remnants.

The sequence of changes in treated cultures was initially a loss of basophilic and other substances and lethal effects on cells dividing at the time of treatment, followed by a delayed cytotoxic action resulting in gross degeneration of the cultures. Prior to the development of delayed toxicity the cell population increased visibly. The collateral cells of a single group often underwent similar degenerative changes concurrently, although the timing of these changes varied from group to group. The delayed cytotoxic effects were associated with reduced nuclear and cytoplasmic basophilia, failure of nuclear replication processes and unusual nuclear lesions. These observations together suggest the possibility that the primary injury to parent cells was related to hereditary material and intensified with successive cell divisions until the derangement became lethal. Intact nuclear membranes appeared to impede the penetration of dextran, presumably of the larger molecules present in " Dextraven ".

The effects upon HeLa cells of administration of " Dextraven " for 4 hours have been described since exposure for this period enabled most of the treated cells to withstand the acute toxicity of dextran and to multiply repeatedly until the manifestation of delayed toxic effects. Longer (6–8 hours) exposure to " Dextraven " had acute cytopathological effects essentially similar to those described. These were, however, more severe. They included a higher proportion of grossly degenerated cells ; a greater loss of basophilic material, especially from nucleoli and cytoplasm, and from mitotic spindles. In many such cultures fixed 24 hours after treatment very little substance stainable by Ehrlich's acid haematoxylin was retained by the cells. These acidophilic cells did not reproduce but viewed *in situ* in the culture vessels they looked morphologically intact (Fig. 4).

Later work has shown that, in addition to the conditions of administration, the response of HeLa cells to " Dextraven " is greatly modified by the nutritive and physiological state of the cells at the time of treatment. Actively dividing, well-fed cells were much more resistant to the toxicity of dextrans than cells taken from exhausted growth or fresh maintenance media. The original observations were made on treated starved cells. These rapidly lost their basophilic content, and presumably other substances, and endured in an apparently unchanged extracted state for an indefinite time. These deficient cells did not recover spontaneously but have been observed, if not too injured, to take up nucleic acids from suitable media. This phenomenon is being investigated.

### DISCUSSION

The cytopathological effects of " Dextraven " resulted from the presence of dextran in this product. The acute initial toxicity was not remarkable in the severe experimental conditions used. But the manifestation of distinctive lethal effects, delayed until treated cells had given rise to several generations, was perhaps more unusual.

The basophilic substances lost from treated cells and their descendants included DNA and RNA. The evidence available implies that dextran-saline also leached out other cell substances. Preliminary cytochemical studies have confirmed the loss of nucleic acids. HeLa cells have been found to be more susceptible to dextran than some other types of cells, for example, Earle's L–929 strain of mouse fibroblasts. Degenerating autolysing cells often tend to lose their nucleic acids. However, the nature and time sequence of pathological changes, including loss of basophilia and nuclear lesions, strongly suggest that the delayed toxicity resulted from injury to cell structures containing nucleic acids and involved in replication processes. The initial lesions of intermitotic cells were not incompatible with survival and reproduction. HeLa cells have been found to tolerate " Dextraven " exposures less severe than those reported here. It is not impossible that small doses of dextran could lead to minute non-lethal biochemical and structural lesions involving nucleic acids. These might result in biological changes among the progeny of injured cells. The carcinogenicity of iron-dextran complex (" Imferon ") established by Richmond (1957, 1959) and Haddow and Horning (1960) may be related in part to the dextran content of this preparation. HeLa cells, derived from a human carcinoma, are not wholly suitable for the investigation of the long term biological effects of dextran. Such studies, as well as others on specific cytological effects of dextran fractions, are being made on normal cells.

### SUMMARY

1. The cytopathological effects of a dextran-saline preparation (" Dextraven ") upon growing HeLa cells are described.

2. During exposure to " Dextraven " dividing cells in the mitotic phases lacking nuclear membranes are killed.

3. Intermitotic cells appear to recover from the initial effects of the agent but, provided they have received a minimum dosage, later die after a number of cell generations.

4. The cytopathological effects described indicate that the delayed toxicity is associated with injury to cell components containing nucleic acids and involved in replication processes.

5. It is suggested that these phenomena may be related to the known carcinogenicity of an iron-dextran complex (" Imferon ").

I am indebted to Mr. G. A. Butcher for his invaluable assistance. The expenses of this work were defrayed from a block grant by the British Empire Cancer Campaign.

### REFERENCES

HADDOW, A. AND HORNING, E. S.—(1960) *J. nat. Cancer Inst.*, **24**, 109.
PEREIRA, H. G. AND KELLY, B.—(1957) *J. gen. Microbiol.*, **17**, 517.
RICHMOND, H. G.—(1957) *Scottish med. J.*, **2**, 169.—(1959) *Brit. med. J.*, i, 947.